# Care and Treatment of Children's Teeth

**Published:** 1898-07

**Authors:** J. W. Paul

**Affiliations:** Minneapolis, Minn.


ARTICLE III.

### CARE AND TREATMENT OF CHILDREN'S TEETH.

J. W. PAUL, D. D. S., MINNEAPOLIS, MINN.

There are many reasons why the teeth of children should have the best care that can be given by parents and family dentists, for I believe that it is just as necessary that every family should have their dentist as physician, and that by having a dentist to look after the teeth of the members of the family, prevent many ills by the prompt relief which he can give.

A man who was suffering from an abscessed tooth was treated by a prominent physician for abscess of the glands of the neck. He was put to quite an amount of expense and pain with only temporary relief. Parents should qualify themselves so as to be able to instruct their children in the care of their teeth, such as the proper sanitary care of the teeth and mouth and the timely removal of the temporary teeth. This last should be with the advice and assitance of the dentist. Many parents cannot distinguish between the temporary and permanent teeth. Especially, they mistake the six year molars for temporary teeth. Temporary teeth are allowed to remain in the mouth long after they should be removed, to the detriment of the permanent set, causing crowding and disallignment and consequent non-articulation. It is sometimes found necessary to remove temporary teeth which have not become loosened by the process of absorption. I have found portions of temporary teeth in the mouths of patients eighteen to twenty years of age wedged in between the permanent teeth. In many children between the ages of two and six I find caries of the teeth. If decay is superficial I apply a remedy to cauterize the surface, which many times is sufficient to prevent further trouble. If the decay is deeper, the enamel and dentin softened, I apply carbolic acid, dry, as well as circumstances will permit, and fill with cement. If the enamel is firm and retaining shape is easily accomplished, I fill with amalgam. If decay has progressed so far as to involve the pulp I sometimes devitalize, remove, and fill the tooth. If the pulp is dead I cleanse cavity, wash out very thoroughly, remove as much of the pulp as possible, apply disinfecting agents, and without attempting to thoroughly fill root-canals, fill the tooth. Much of our work with children must be palliative, not to attempt long operations. There should be no gold fillings in temporary teeth and few in the first years of permanent teeth. A dentist to be successful with very young patients must have tact and kindness of heart. A child learns to discriminate between professional urbanity and disinterested sympathy and gentleness. If the confidence of the child is once gained it will bear a surprising amount of pain. I think the mistake is made of trying to do too much at one sitting, tiring the child and causing it to lose courage by the long endurance. Better short operations and often. A child should be taught the use of the brush as early as possible, and continually admonished to use it till it becomes a necessity. I believe an examination of the teeth of the children of the public schools as often as once in a year is as necessary as the examination of the eyes.

As dentists we have a duty to perform in advising, admonishing and aiding in every way that we can till these little sufferers will learn to appreciate the benefit we do for them.—*Review.*

